# Lipedema and adipose tissue: current understanding, controversies, and future directions

**DOI:** 10.3389/fcell.2025.1691161

**Published:** 2025-11-06

**Authors:** Atefeh Rabiee

**Affiliations:** Department of Pharmaceutical Sciences, Thomas J. Long School of Pharmacy, University of the Pacific, Stockton, CA, United States

**Keywords:** lipedema, adipose tissue, macrophages, fibrosis, inflammation, biomarkers

## Abstract

Lipedema is a chronic disorder characterized by the symmetrical accumulation of subcutaneous adipose tissue, predominantly affecting women. Despite increasing recognition, the pathophysiological mechanisms underlying adipose tissue dysfunction in lipedema remain incompletely understood. This mini review combines current knowledge about adipose tissue biology in lipedema, highlighting recent discoveries, ongoing controversies, and future research directions. A comprehensive literature review was conducted focusing on adipose tissue-related research in lipedema with emphasis on pathophysiological mechanisms, cellular composition, and therapeutic implications. Recent studies reveal that lipedema adipose tissue exhibits distinct characteristics, including M2 macrophage predominance, stage-dependent adipocyte hypertrophy, progressive fibrosis, and altered lymphatic/vascular function. The inflammatory profile differs markedly from obesity, with an anti-inflammatory M2-like macrophage phenotype rather than the pro-inflammatory M1 response seen in classic obesity. Emerging evidence suggests lipedema may represent a model of “healthy” subcutaneous adipose tissue expansion with preserved metabolic function despite increased adiposity. Current research proposes menopause as a critical turning point, driven by estrogen receptor imbalance and intracrine estrogen excess. Lipedema represents a unique adipose tissue disorder distinct from obesity, characterized by specific cellular and molecular signatures. Current research gaps include the need for validated biomarkers, standardized diagnostic criteria, and targeted therapeutics. Future research should focus on elucidating the molecular mechanisms driving adipose tissue dysfunction and developing precision medicine approaches.

## 1 Introduction

Lipedema is a chronic, progressive disorder affecting an estimated 10%–20% of women worldwide, characterized by the bilateral and symmetrical accumulation of subcutaneous adipose tissue (SAT) in the extremities, particularly the legs and arms, while typically sparing the hands and feet (Poojari et al., 2022). Despite its high prevalence, lipedema remains frequently misdiagnosed as obesity or lymphedema, leading to significant patient suffering, delayed treatment, and psychosocial distress (van la Parra et al., 2023). The condition often manifests or exacerbates during periods of hormonal fluctuation, such as puberty, pregnancy, and menopause, underscoring a strong endocrine influence in its pathogenesis (Al-Ghadban et al., 2021). Patients experience disproportionate fat deposits that are resistant to conventional weight loss methods, accompanied by symptoms like pain, tenderness, easy bruising, and in advanced stages, mobility limitations (van la Parra et al., 2023).

The disorder progresses through three recognized stages: Stage I involves smooth skin with enlarged subcutaneous fat; Stage II features skin dimpling and nodularity; and Stage III presents with large fat lobules and severe tissue deformation (Rasmussen et al., 2022). Beyond physical symptoms, lipedema can lead to emotional challenges, including body image issues and depression, highlighting the need for holistic management approaches. Recent advances in adipose tissue research have illuminated fundamental differences between lipedema fat and that seen in obesity or normal adipose expansion. This preservation may stem from the subcutaneous nature of the fat accumulation, which avoids the visceral fat-related risks associated with metabolic syndrome. Historical perspectives trace lipedema back to descriptions in the 1940s, but only in the last decade has it gained traction as a distinct entity, with international societies forming to standardize care and research efforts (Cifarelli, 2025). Epidemiological data suggests genetic predispositions, with familial clustering in up to 60% of cases, though specific genes remain elusive (Mortada et al., 2025).

**FIGURE 1:**
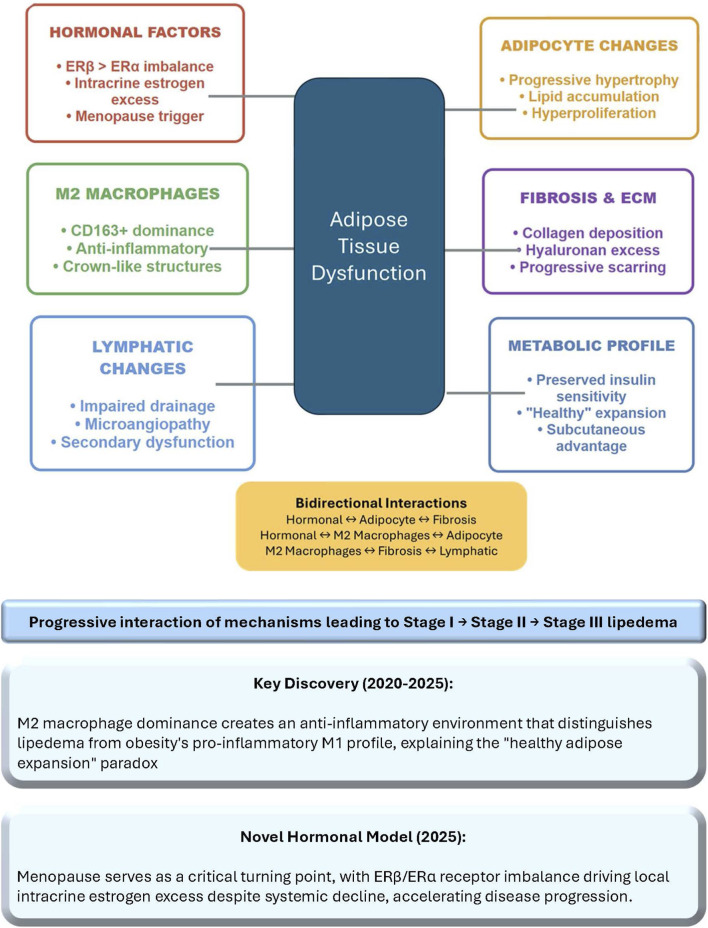
Pathophysiological mechanisms in lipedema. The diagram illustrates the complex network of bidirectional interactions between six key pathophysiological mechanisms contributing to adipose tissue dysfunction in lipedema. The central navy box represents the convergence of all mechanisms on adipose tissue dysfunction, the core pathological process driving disease progression from Stage I to Stage III. Potential bidirectional interactions are summarized in yellow box. Key discoveries from 2020 to 2025 research include the predominance of anti-inflammatory M2 macrophages distinguishing lipedema from obesity’s pro-inflammatory profile, and the 2025 hormonal model identifying menopause as a critical accelerator through ERβ/ERα imbalance and local estrogen overproduction. ECM, extracellular matrix; ER, estrogen receptor.

This mini-review bring together current knowledge on adipose tissue biology in lipedema. It examines cellular composition, molecular mechanisms, and therapeutic implications, while addressing ongoing controversies and outlining future research directions. By integrating multi-omics data, histological analyses, and clinical observations, we aim to provide a comprehensive overview that bridges basic science with clinical practice (Straub et al., 2025). The review also incorporates insights from emerging models, such as the role of immune cells and hormonal dysregulation, to foster a deeper understanding of this understudied condition. The complex network of bidirectional interactions between these key pathophysiological mechanisms is visualized in Figure 1, highlighting how hormonal factors, M2 macrophage polarization, adipocyte dysfunction, progressive fibrosis, lymphatic impairment, and preserved metabolic health converge to drive central adipose tissue dysfunction in lipedema.

## 2 Pathophysiological mechanisms of adipose tissue dysfunction in lipedema

The pathophysiology of lipedema involves complex interactions between adipocytes, immune cells, extracellular matrix (ECM), and hormonal factors, leading to dysfunctional adipose tissue expansion. Unlike obesity, where visceral fat accumulation drives metabolic syndrome, lipedema primarily affects subcutaneous depots with distinct molecular signatures (Felmerer et al., 2020; Duhon et al., 2022; Wolf et al., 2022). These signatures include altered gene expression profiles related to lipid metabolism, inflammation, and fibrosis, which contribute to the unique clinical presentation. Recent longitudinal observations indicate that early intervention might alter disease trajectory, but mechanistic details are still emerging (Dadras et al., 2017).

### 2.1 Cellular composition and macrophage polarization

A hallmark discovery in lipedema research is the unique inflammatory profile of affected adipose tissue, dominated by anti-inflammatory M2 macrophages expressing high levels of CD163, in stark contrast to the pro-inflammatory M1 macrophages prevalent in obesity (Straub et al., 2025). This M2 predominance may arise from chronic low-level exposure to lipopolysaccharide, interleukin 10, or estrogens, promoting lipid accumulation and adipocyte hypertrophy (Felmerer et al., 2020; Duhon et al., 2022; Wolf et al., 2022). Immunohistochemical studies confirm this M2 bias is region-specific, evident in thigh tissue but absent in abdominal regions, suggesting localized triggers (Kruppa et al., 2023).

The functional role of M2 macrophages extends to modulating ECM remodeling and vascular function, potentially explaining the resistance to weight loss. Emerging evidence proposes that complement system dysregulation, with upregulated classical pathway components like C1q, induces M2 polarization, further enhancing adipogenesis (Bohlson et al., 2014). Therapeutic modulation, such as with phosphatidylinositol-3 kinase γ inhibitors, has shown promise in shifting M2 towards M1 phenotypes, normalizing adipogenic potential in preclinical models (Felmerer et al., 2020). This challenges traditional inflammation paradigms and positions lipedema as a model for anti-inflammatory adipose expansion. Additionally, studies indicate that M2 macrophages may interact with adipose stem cells to perpetuate tissue growth, creating a self-sustaining cycle of hypertrophy (Pagani et al., 2024). Single-cell analyses have further revealed subpopulations of these macrophages with varying secretory profiles, influencing local tissue homeostasis (Pagani et al., 2024).

### 2.2 Adipocyte biology and tissue remodeling

Histological analyses reveal stage-dependent adipocyte hypertrophy in lipedema, with cell sizes increasing progressively from Stage I to III, particularly in affected thighs (Kruppa et al., 2023). In early stages, adipocytes show mild enlargement without significant metabolic impairment, but advanced stages feature pronounced hypertrophy, crown-like structures of necrotizing adipocytes surrounded by CD68^+^ macrophages, and proliferation of adipose-derived stem cells (Wolf et al., 2022; Maria Ernst et al., 2024). Transcriptomic profiling identifies over 4,000 differentially expressed genes involved in lipid metabolism, cell proliferation, and cycle regulation, favoring hyperproliferation and fibrosis (Streubel et al., 2024).

Tissue remodeling in lipedema balances degenerative and regenerative processes, with increased commitment of preadipocytes to differentiation, potentially driven by stromal vascular fraction alterations (Kruppa et al., 2023). This dynamic environment leads to dysfunctional fat depots resistant to lipolysis, contributing to the clinical phenotype. Multi-omics approaches further reveal upregulated mitochondrial organization and aerobic respiration, suggesting adaptive mechanisms that preserve function amid expansion (Straub et al., 2025). Recent scRNA-seq data highlight adipocyte heterogeneity, with distinct clusters showing varied expression of fibrotic genes (Pagani et al., 2024).

### 2.3 Fibrosis and extracellular matrix remodeling

Progressive fibrosis is a core feature of lipedema adipose tissue, with interstitial collagen accumulation correlating with disease severity, explaining the characteristic firm texture and resistance to weight loss (Kruppa et al., 2023). ECM remodeling involves sodium concentration alterations, collagen deposition, and glycocalyx disruptions, including proteoglycans and glycosaminoglycans, leading to microangiopathy and further scarring (Drygalski et al., 2024).

In advanced stages, fibrosis may impair lymphatic drainage, exacerbating edema-like symptoms, though distinct from primary lymphedema (Felmerer et al., 2020; Duhon et al., 2022; Wolf et al., 2022). Endothelial cell changes in capillaries of affected adipose tissue, such as altered glycocalyx and increased permeability, contribute to this process (Felmerer et al., 2020). Targeting fibrotic pathways, like transforming growth factor-beta signaling, could offer therapeutic avenues to halt progression. Recent findings also highlight hyaluronan in the ECM promoting fat cell growth and collagen buildup, underscoring the need for anti-fibrotic strategies (Drygalski et al., 2024). Studies using transcriptomics have identified key dysregulated networks in ECM genes, providing targets for intervention (Streubel et al., 2024).

### 2.4 Hormonal mechanisms and estrogen signaling

**FIGURE 2:**
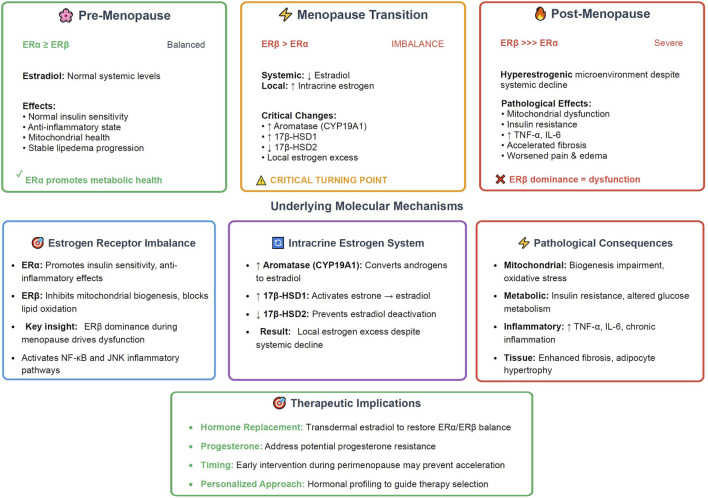
Menopause as critical turning point - The estrogen receptor model in lipedema (2025). The timeline illustrates the three distinct phases of hormonal changes that influence lipedema progression, with menopause serving as the critical accelerator. Pre-menopause (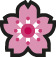, ∼40–45 years) shows balanced ERα ≥ ERβ receptor expression with normal systemic estradiol levels, maintaining insulin sensitivity, anti-inflammatory state, and stable disease progression. Menopause transition (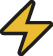, 45–55 years) represents the critical turning point characterized by ERβ > ERα receptor imbalance coinciding with declining systemic estradiol but increasing local intracrine estrogen production through upregulated aromatase (CYP19A1) and 17β-HSD1, combined with decreased 17β-HSD2 expression. Post-menopause (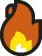, 55+ years) features severe ERβ >>> ERα dominance creating a hyperestrogenic microenvironment despite systemic estradiol decline, leading to mitochondrial dysfunction, insulin resistance, inflammatory cytokine elevation (TNF-α, IL-6), accelerated fibrosis, and worsened clinical symptoms. The underlying molecular mechanisms section details the estrogen receptor functional differences, intracrine estrogen enzymatic pathways, and resulting pathological consequences. Therapeutic implications highlight hormone replacement strategies targeting ERα/ERβ balance restoration, progesterone supplementation, and the importance of early perimenopause intervention through personalized hormonal profiling. This model positions menopause as the pivotal event driving lipedema acceleration through local estrogen excess rather than deficiency. ER, estrogen receptor; HSD, hydroxysteroid dehydrogenase; TNF-α, tumor necrosis factor-alpha; IL-6, interleukin-6.

A groundbreaking 2025 model positions menopause as a critical turning point in lipedema progression, driven by estrogen receptor (ER) imbalance—predominance of ERβ over ERα—intracrine estrogen excess, and adipose tissue dysfunction (Pinto da Costa Viana et al., 2025). During menopause, systemic estradiol decline suppresses ERα signaling, which normally promotes insulin sensitivity and anti-inflammatory effects, while enhancing ERβ activity that inhibits mitochondrial biogenesis and lipid oxidation (Pinto da Costa Viana et al., 2025). This shift induces mitochondrial glitches, insulin resistance, and a pro-inflammatory milieu via NF-κB and JNK pathways, elevating cytokines like TNF-α and IL-6 (Pinto da Costa Viana et al., 2025). Temporal progression of this hormonal model, the transition from balanced ERα/ERβ signaling in pre-menopause through the critical turning point of menopause transition to the pathological ERβ dominance in post-menopause, along with the underlying molecular mechanisms and therapeutic implications is illustrated in Figure 2.

Intracrine estrogen excess arises from upregulated aromatase (CYP19A1) and 17β-HSD1, converting precursors to active estradiol locally, while 17β-HSD2 suppression prevents deactivation (Pinto da Costa Viana et al., 2025). This hyperestrogenic microenvironment perpetuates adipocyte hypertrophy, fibrosis, and immune dysregulation (Bicca, 2024). ERα enhances lipogenesis and angiogenesis in gynoid regions, while ERβ dominance during hormonal transitions exacerbates dysfunction (Bicca, 2024).

Clinical implications include worsened pain, fluid retention, and fat resistance post-menopause, suggesting hormone-modulating therapies like transdermal estradiol or progestins as potential interventions (Bicca, 2024). Progesterone resistance may compound this, linking lipedema to broader gynecological conditions (Tomada, 2025). Recent narrative reviews emphasize the role of hormonal fluctuations in disease onset and progression, calling for more longitudinal hormonal profiling (Tomada, 2025).

## 3 Current controversies and schools of thought

### 3.1 Lymphatic vs. adipose tissue disease

Lipedema is defined as a chronic disorder involving symmetrical subcutaneous adipose tissue accumulation, primarily in the extremities, with associated pain and bruising but preserved lymphatic function in early stages. Lymphedema, in contrast, is characterized by lymphatic system impairment leading to fluid accumulation and swelling, often unilateral and involving the hands/feet. Lipolymphedema (or phlebolymphedema) represents a combined condition where lipedema progresses to include secondary lymphatic or venous dysfunction, resulting in overlapping symptoms such as edema and fibrosis (Dean et al., 2020; Lomeli et al., 2024). Lipedema is primarily an adipose tissue disorder, with potential secondary lymphatic involvement that may contribute to symptom severity. While the condition shares some characteristics with lymphedema, such as fluid accumulation and fibrosis, a notable distinction is that many experts favor the idea of a primary adipose pathology with secondary lymphatic involvement. This perspective is supported by research that focuses on the unique biology of lipedema fat, (Lomeli et al., 2024), even though biomarkers like platelet factor 4 (PF4) have been found that are linked to lymphatic issues (Ma et al., 2020). The classification is further complicated by the fact that lipedema can exist alongside other conditions, like phlebolymphedema (Dean et al., 2020; Lomeli et al., 2024). To reconcile these conflicting views, some researchers have proposed a hybrid model in which lymphatic dysfunction is not the direct cause of the disease but instead amplifies the underlying pathology of the adipose tissue. This hypothesis is supported by imaging studies that have revealed microvascular changes, providing a more nuanced understanding of the complex interplay between the lymphatic and adipose systems in lipedema (Buso et al., 2019; Rasmussen et al., 2022).

### 3.2 Primary vs. secondary pathophysiology

One of the most significant and unresolved questions in lipedema research is whether the various changes in the body—the hormonal shifts, lymphatic issues, and adipose tissue abnormalities—are the cause or the result of the disease. The precise interplay between these systems remains unclear, with some researchers arguing that lymphatic dysfunction is the initial problem (Rasmussen et al., 2022), while others believe that the primary issue lies within the adipose tissue itself (Felmerer et al., 2020; Duhon et al., 2022; Wolf et al., 2022). Clarifying this temporal sequence is a top research priority. Understanding the causal chain would be invaluable, as it could inform preventive strategies. For example, some mechanistic models suggest a “vicious cycle” where the initial enlargement of fat cells, known as adipose hypertrophy, is the primary event, which then leads to secondary lymphatic impairment. This secondary impairment could further worsen the fluid retention and tissue changes, creating a feedback loop that drives the disease’s progression. Resolving this debate is crucial for developing therapies that target the root cause of lipedema rather than just its symptoms (Grewal et al., 2025).

### 3.3 Diagnostic criteria and disease staging

Diagnosing lipedema relies on clinical observations including female predominance, disproportionate fat accumulation, hormonal connections, easy bruising, and pain. However, experts disagree on the importance of family history and edema as diagnostic criteria, with calls for consensus guidelines integrating imaging and biomarkers (Bertsch et al., 2020; Faerber et al., 2024).

### 3.4 The ‘healthy adipose expansion’ debate

#### 3.4.1 Metabolic characteristics

Lipedema may represent “healthy” SAT expansion, with lower android-to-gynoid fat ratios and preserved insulin sensitivity compared to body mass index (BMI)-matched controls (Nankam et al., 2022). Despite elevated BMI, diabetes prevalence is low (5%) and dyslipidemia (7%), with studies showing 48% greater insulin sensitivity in obese lipedema patients (Nankam et al., 2022). Lower HbA1c (5.55% vs. 6.73%) indicates better glucose metabolism, though higher cholesterol and LDL-C suggest mixed lipid risks (Nankam et al., 2022). Elevated adiponectin may protect against metabolic harm, countering inflammation and oxidative stress markers like TNFSF14 and malondialdehyde (Nankam et al., 2022). Comparative studies with population-matched controls further support lower cardiometabolic risk, though long-term outcomes require monitoring (Tomada, 2025).

#### 3.4.2 Regional differences in adipose tissue function

Comparative analyses show decreased lymphatic/vascular gene expression and increased fibrosis/inflammation genes in thigh vs. abdominal SAT (Kruppa et al., 2023). This heterogeneity explains selective depot involvement and metabolic preservation, as thigh expansion avoids visceral risks (Cifarelli, 2025). Such differences underscore that not all adipose accumulation is detrimental, informing targeted therapies. Regional variations also highlight potential for depot-specific interventions, like localized anti-fibrotic agents. Transcriptomic data reveal depot-specific immune signatures, with thigh tissue showing more M2-like profiles (Lomeli et al., 2024).

## 4 Current research gaps

### 4.1 Biomarker development

Validated biomarkers are absent, impeding diagnosis. Metabolomics identify pyruvic acid and glutamic acid as candidates, with multi-omics panels showing promise for 80% accuracy (Kempa et al., 2023). Larger cohorts are essential for validation, and platelet factor 4 has emerged as a potential lymphatic biomarker (Ma et al., 2020). The lack of specific biomarkers delays diagnosis and complicates monitoring disease progression and treatment efficacy. Recent studies have explored circulating microRNAs and inflammatory markers like adiponectin, but reproducibility across diverse populations remains low (Cione et al., 2024). Without these, misdiagnosis rates could remain as high as 80%, exacerbating patient burden (Mortada et al., 2025).

### 4.2 Therapeutic target identification

Options are limited; ketogenic diets show symptom relief, but mechanisms require elucidation (Sanlier and Baltacı, 2025). 2024 guidelines endorse Mediterranean or ketogenic approaches, yet targeted drugs for pathways like mitochondrial organization are lacking (Faerber et al., 2024). Current treatments offer symptomatic relief but fail to address underlying pathology, while surgical interventions face questions about long-term efficacy and risks (Mortada et al., 2025). Surgical interventions like liposuction provide volume reduction, but long-term efficacy and risks, such as lymphatic damage, are debated (Mortada et al., 2025). Emerging targets include estrogen modulators and anti-fibrotic agents, but preclinical data is limited.

### 4.3 Natural history and disease progression

Lipedema’s trajectory—onset triggers, progression rates, and outcomes—is poorly mapped. Longitudinal studies tracking biomarkers and responses are crucial (Carvalho, 2024). The natural history involves variable progression, with some patients stable for years while others advance rapidly post-hormonal events. Lack of prospective cohorts means uncertainty in risk factors, such as BMI influence or comorbidity development. Addressing this gap could inform preventive strategies and improve prognostic models for personalized care.

## 5 Future research directions

### 5.1 Precision medicine approaches

Personalized strategies based on molecular profiles, stages, and responses are key. AI and pharmacogenomics could optimize diagnostics and interventions (Cifarelli, 2025). Genetic profiling may enable tailored hormone therapies. AI integration, such as using large language models like GPT-4 for consultation assistance, shows promise in enhancing diagnostic accuracy and patient education (Leypold et al., 2024). Future applications include machine learning algorithms analyzing imaging and genetic data to predict treatment responses, reducing trial-and-error in therapy selection. Pharmacogenomics could identify variants affecting estrogen metabolism, guiding targeted interventions (Chen et al., 2025). Collaborative platforms for data sharing will accelerate precision approaches.

### 5.2 Mechanistic studies

Future mechanistic studies should elucidate M2 dominance, hormone-adipose links, and fibrosis initiation using single-cell RNA sequencing and spatial transcriptomics (Straub et al., 2025). These tools promise insights into cellular interactions. Preliminary scRNA-seq in 2024 uncovered adipocyte heterogeneity in lipedema, identifying subpopulations with distinct gene expressions related to fibrosis and inflammation (Pagani et al., 2024). Expanding to larger cohorts could map dynamic changes over disease stages. Spatial transcriptomics may reveal microenvironmental niches driving pathology, informing targeted therapies (Liu et al., 2022). Integrating with proteomics will provide a further holistic view of protein interactions.

### 5.3 Therapeutic development

Future therapeutic development should investigate ketogenic diets’ anti-inflammatory and lymphatic benefits, with evidence of BMI reductions (4.23 kg/m^2^) and pain relief over weeks (Verde et al., 2023; Sanlier and Baltacı, 2025). Future targets include ER signaling, macrophage polarization, and fibrosis (Pinto da Costa Viana et al., 2025). Low-carb diets have demonstrated reductions in subcutaneous fat and cytokines in 2024 studies (Lundanes et al., 2024). Emerging trials for tirzepatide and other metabolic agents could bridge gaps, with focus on randomized designs (Sanlier and Baltacı, 2025).

### 5.4 Technology integration

Technology integration should incorporate AI for diagnosis, wearables for monitoring, and telemedicine for access, integrated with biomarkers for early intervention (Bouillon et al., 2023; Arun et al., 2024). Digital health tools could revolutionize care. Wearables tracking limb volume and activity show promise in lymphedema management, adaptable to lipedema for real-time progression monitoring (Bouillon et al., 2023). Telemedicine platforms enable remote consultations, improving access in underserved areas (Mortada et al., 2025). AI-driven apps for symptom tracking could personalize interventions, with 2025 trends emphasizing integration with remote patient monitoring (Arun et al., 2024).

## 6 Discussion

Key discoveries include M2 macrophage predominance, ‘healthy adipose expansion’ hypothesis, and novel hormonal mechanisms (Nankam et al., 2022; Grewal et al., 2025; Pinto da Costa Viana et al., 2025). The ‘healthy adipose expansion’ hypothesis, while promising, requires further longitudinal data to clarify its metabolic implications (Kruppa et al., 2023).

However, significant translational gaps persist, hindering the application of these findings to clinical practice. The absence of validated biomarkers continues to fuel high misdiagnosis rates which delays appropriate care and exacerbates patient suffering (Ma et al., 2020). Limited therapeutic options remain predominantly symptomatic, relying on compression garments, manual lymphatic drainage, and surgical liposuction, none of which address the underlying pathophysiology. Emerging nutritional interventions, like ketogenic diets, show promise in reducing inflammation and improving symptoms, but require rigorous mechanistic elucidation through controlled trials (Verde et al., 2023; Sanlier and Baltacı, 2025).

The field stands at a pivotal juncture where basic science insights are converging with therapeutic possibilities. Priority areas for immediate investment include large-scale biomarker validation studies, incorporating multi-omics to develop diagnostic panels with high sensitivity and specificity (Straub et al., 2025). Mechanistic investigations using advanced tools like single-cell RNA sequencing will further dissect cellular dynamics, while standardized outcome measures for clinical trials are essential to evaluate interventions robustly.

Ultimately, success in bridging these gaps will demand sustained funding, cross-disciplinary collaboration, and amplified patient advocacy to ensure scientific advances translate into tangible clinical benefits. This evolution promises improved patient outcomes and broader implications for adipose biology, potentially informing treatments for related conditions like obesity and lymphedema.
